# Review on Synthesis of Silica-Based Hybrid Sorbents and Their Application in Radionuclide Separation and Removal via Chromatographic Technique

**DOI:** 10.3390/toxics13040319

**Published:** 2025-04-19

**Authors:** Xiangbiao Yin, Fan Wang, Qi Zheng, Shunyan Ning, Lifeng Chen, Yuezhou Wei

**Affiliations:** 1 School of Nuclear Science and Technology, University of South China, 28 Changsheng West Road, Hengyang 421001, China; yinxb@usc.edu.cn (X.Y.); wangfan0911@stu.edu.cn (F.W.); zq@stu.usc.edu.cn (Q.Z.); chenlf@usc.edu (L.C.); 2 Key Laboratory of Advanced Nuclear Energy Design and Safety, Ministry of Education, University of South China, 28 Changsheng West Road, Hengyang 421001, China; 3 School of Nuclear Science and Engineering, Shanghai Jiao Tong University, 800 Dong Chuan Road, Shanghai 200240, China

**Keywords:** silica-based sorbents, radionuclides, HLLW, separation, extraction chromatography

## Abstract

The efficient separation and removal of key nuclides is important for the nuclear fuel cycle from the aspects of radioactivity reduction and potential resource recycling. The urgent objective is to design and develop functional materials for the separation and removal of important nuclides. Porous silicon-based adsorbents are considered highly advantageous materials for separating and removing radioactive nuclides in complex environments due to their excellent mechanical properties, high porosity, and functionalization ability. In this review, we compiled the applications of porous silica-based materials in recent years in the separation and removal of key nuclides, such as actinides, lanthanides, strontium, cesium, iodine, and platinum group metals; discussed their separation and removal performances; analyzed the constitutive relationship between key radionuclides and porous silica-based adsorbents; and systematically described the properties and mechanisms of different types of porous silica-based adsorbents. This article aims to provide some ideas for the design of an advanced separation process in the nuclear fuel cycle.

## 1. Introduction

**Figure 1:**
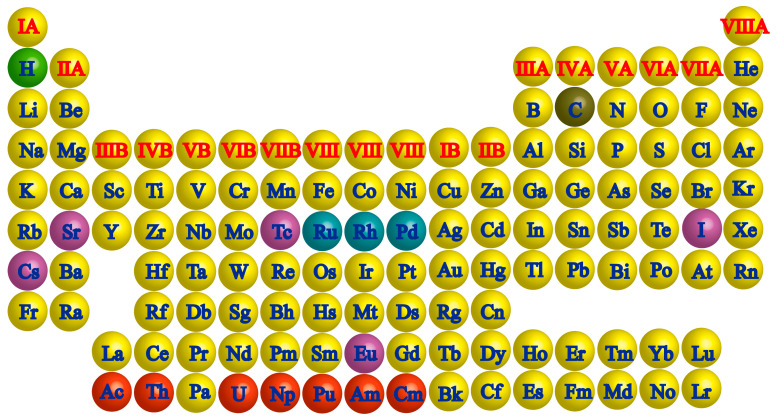
Key radioactive nuclides in the periodic table of elements (marked with different spherical colors).

With the proposal of “carbon peak and carbon neutrality” (on 22 September 2020, China announced that it aims to peak its carbon dioxide emissions before 2030 and strive to achieve carbon neutrality before 2060), China is expected to establish more nuclear power plants to meet the needs of energy structure adjustment [1,2]. The management and efficient disposal of radioactive waste in the process of nuclear energy utilization is one of the important factors contributing to the development of nuclear power. The radionuclides in the high-level liquid waste (HLLW) generated by the plutonium and uranium recovery-by-extraction (PUREX) process are classified as minor actinides (MAs) [3,4], long-term fission products (mainly iodine) [5,6], the middle-to-long-term heat-release radioactive elements (mainly Sr and Y) [7,8], platinum group metals (PMGs) [9,10], and other fission products according to their special properties, e.g., chemical properties, half-life, decay type and energy, mobility, biological affinity, and impact on HLLW treatment (Figure 1).

Different functional materials and corresponding processes, including organic and inorganic, have been previously developed to separate the radionuclides, such as CMPO [11], Cyanex301 [12], TRPO [13], and TODGA [14], for their co-extraction of minor actinides and lanthanides, the N-containing ligands BTPs and their derivatives for the selective separation of minor actinides, DtBuCH18C6 for the extraction of Sr, Calix for the extraction of Cs, and other natural sorbents (note: all the abbreviations of the ligands and extractants mentioned in the manuscript have been given full names and chemical structures in Table 1). To overcome the problems existing in liquid–liquid extraction (such as the third phase caused by emulsification, multistage extraction to achieve good decontamination effect, large consumption amount of organic solvents, low extraction ability caused by poor solubility of the ligands or extraction complex, and so on), extraction chromatography based on the solid–liquid sorption method had been proposed and has attracted more and more attention [15,16].

Compared with liquid–liquid extraction, extraction chromatography has the following advantages: (1) the lowest utilization rate of organic solvents and less organic waste accumulation [17,18]; (2) a clear operation environment with less toxicity and smells caused by organic solvents [19]; and (3) compact equipment. In the practical application of chromatographic separation and the removal of radioactive nuclides, the most concerning issues are how to replace fillers, unblock column blockages, and avoid leakage caused by overpressure. The key is to prepare solid adsorption materials that are suitable for packed or adsorption towers and can operate continuously. Efficient adsorbents for radioactive nuclide separation should have good chemical and mechanical stability, excellent pore structure, and uniform particle size. The adsorbents mentioned in this study are usually prepared by impregnating or grafting the above-mentioned materials (including organic and inorganic materials) onto appropriate carriers, such as Amberlite ^®^ XAD series (based on styrene divinylbenzene) [20]. But some shortcomings, such as low sorption capacity, premature penetration, and serious tailing, are frequently found in the case of XAD-based materials.

**Figure 2:**
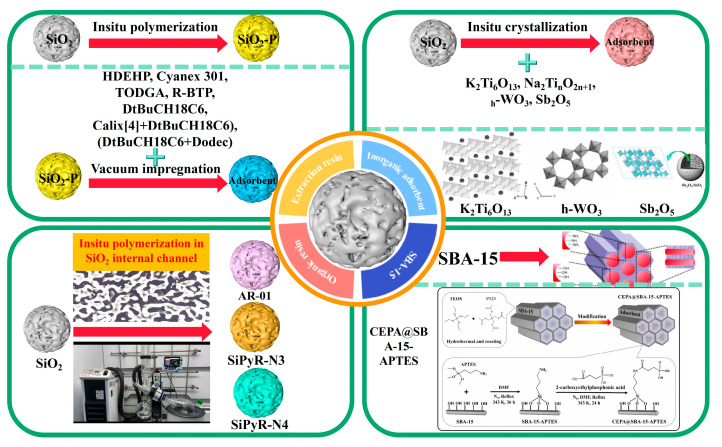
Common porous silicon-based materials synthesized for the adsorption of radionuclides [26,27,28,29,30,31,32,33].

A macroporous silica-based support (SiO_2_) was typically developed with a porosity (porosity is a dimensionless parameter that represents the ratio of pore volume to total volume in a material, usually expressed as a percentage) of about 70%, average pore size of 50 nm or even 600 nm, and average particle size of about 60 μm, which is about 1/10 of that of commercial resin [21]. It is evident that traditional inorganic carriers, including carbon-based material [22], zeolite [23], and diatomaceous earth [24], exhibit inherent defects. These defects are mainly characterized as an irregular shape, low mechanical strength, and small pore size. However, silica-based carriers have excellent mechanical properties, high porosity, and a small particle size, making them an ideal carrier for preparing composite adsorbents. Furthermore, they can be modified through the copolymerization of styrene and divinylbenzene to prepare an organic–inorganic composite carrier (SiO_2_-*P*) to increase the affinity with organic ligands, where “*P*” refers to the copolymer [25]. Due to the porous structure of SiO_2_ and SiO_2_-*P*, adsorbents prepared with them exhibit rapid adsorption kinetics, low column pressure, and high adsorption efficiency. This study reported various organic or inorganic adsorbents, such as TODGA/SiO_2_-*P*, CMPO/SiO_2_-*P*, R-BTP/SiO_2_-*P*, DtBuCH18C/SiO_2_-*P*, etc., for the separation of key nuclides. And different separation experiments were designed based on different adsorbents and target nuclides, which will be explained in detail in the next section (Figure 2).

## 2. Preparation of SiO_2_- or SiO_2_-*P*-Based Adsorbents

**Figure 3:**
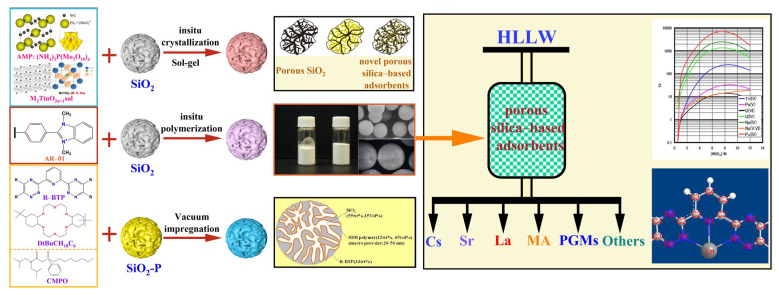
Preparation of porous silicon-based adsorbents and their application in the fields of separation and recovery of various radioactive nuclides [28,32,40,41,42].

Firstly, the stability of SiO_2_-*P*, in 0–3 M HNO_3_ solution under γ irradiation, was studied. It was found that the TOC (total organic carbon) in the solution from polymer degradation was negligibly small. There was little leakage of carbon from SiO_2_-*P* particles by γ irradiation up to about 1 MGy [34]. The above results indicate that SiO_2_-*P*, as a very stable carrier, can be used for the separation of radioactive nuclides. Different methods have been developed for SiO_2_- or SiO_2_-*P*-based sorbent preparation, including vacuum impregnation [35], in situ polymerization [36], the sol–gel method [37], hydrothermal method [38], and chemical grafting [39]. Most organic ligands with high solubility in low-boiling organic solvents, such as CH_2_Cl_2_, CH_3_OH, CH_2_CH_2_OH, etc., can be dissolved in specific organic solvents and then immersed into porous SiO_2_-*P* under vacuum. The composite sorbents, such as HDEHP/SiO_2_-*P*, Cyanex301/SiO_2_-*P*, CMPO/SiO_2_-*P*, TODGA/SiO_2_-*P*, R-BTP/SiO_2_-*P*, and DtBuCH18C6/SiO_2_-*P*, were all prepared by dissolving the corresponding ligands in CH_2_Cl_2_. After thorough mixing, the ligands were impregnated into the SiO_2_-*P* matrix under vacuum, with the temperature precisely controlled at approximately 39 °C. Also, two or more ligands including modifiers (e.g., TBP, TOA, octanol, dodecane, etc.) can be also added into the solvents together to realize synergistic adsorption or improve the hydrophobicity or other properties, such as (Calix[4]+DtBuCH18C6)/SiO_2_-*P*, (DtBuCH18C6+TBP)/SiO_2_-*P*, (DtBuCH18C6+Dodec)/SiO_2_-*P*, (DtBuCH18C6+Oct)/SiO_2_-*P*, (Calix[4]+Dodecanol)/SiO_2_-*P*, and (Calix[4]+dodecanol+DBS)/SiO_2_-*P*. In addition, some inorganic adsorbents can be prepared using the sol–gel method, such as AMP (ammonium molybdate)/SiO_2_, K_2_Ti_6_O_13_/SiO_2_, and Na_2_TinO_2n+1_/SiO_2_. The above prepared sorbents can be used for the separation and recovery of various radionuclides, including Cs, Sr, minor actinides, etc. (Figure 3).

## 3. Separation and Removal of Key Radioactive Nuclides

### 3.1. Actinide Separation

During the PUREX process, as U and Pu are separated, increased attention is directed toward the separation of minor actinides, particularly the mutual group separation of trivalent minor actinides (MA(III)) and trivalent lanthanides (Ln(III)) [43,44]. Although MA (III) accounts for only about 0.1% of spent fuel, it is crucial for reducing the long-term toxicity of radioactive waste [45]. There are two main strategies for separating and recovering actinide elements. One would be the co-extraction of MA(III) and Ln(III) first from higher-acid HLLW, followed by their mutual separation in low-acid solution. The other is the direct separation of MA(III) from higher-acid HLLW, which is very challenging [46,47,48]. During the past twenty years, several ligands have been developed and extensively studied, such as TODGA, CMPO, HDEHP, Cyanex301, and R-BTP and its derivatives, R-BTBP and R-BTPhen. The above-mentioned silicon-based adsorbents were prepared using a simple vacuum impregnation method. Table 2 provides a summary and comparison of the adsorption and separation of minor actinide elements using silicon-based adsorbents.

CMPO/SiO_2_-*P* and TODGA/SiO_2_-*P* exhibited good adsorption toward MA(III) and Ln(III) in 3 M HNO_3_ solution, while desorption was efficiently performed using lower-acid solution or water or DTPA (the adsorption mechanism is detailed in Equations (1) and (2)). But there are also some differences between them. TODGA/SiO_2_-*P* exhibited selective adsorption toward Sr(II) but poor adsorption toward Mo(IV) in 3 M HNO_3_ solution, while CMPO/SiO_2_-*P* was just inversed [49]. Furthermore, by adding DTPA into the eluent solution from the TODGA/SiO_2_-*P* column and adjusting the acidity to 3 M, CMPO/SiO_2_-*P* can separate the metal ions into (Pd+Sr)/(MA+Ln)/Zr groups, which provides a new idea for Sr(II) separation. The (MA+Ln) groups can be realized for their mutual group separation in lower-acid solution using many other materials, such as R-BTP/SiO_2_-*P*, HDEHP/SiO_2_-*P*, and Cyanex301/SiO_2_-*P*. The adsorption of HDEHP/SiO_2_-*P* toward MA(III) and Ln(III) decreased with HNO_3_ concentration due to the H^+^ competition effect, and it exhibited preferential adsorption of Ln(III) over MA(III). Cyanex301/SiO_2_-*P* exhibited good adsorption selectivity toward Am(III) over Ln(III) in a mildly acidic medium, such as pH = 4, and the adsorbed Am(III) can be desorbed using 0.1 M HNO_3_. Typically, it is worth noting that the purity of Cyanex 301 has a significant impact on the separation. By combining CMPO/SiO_2_-*P* or TODGA/SiO_2_-*P* with HDEHP/SiO_2_-*P* or Cyanex301/SiO_2_-*P*, MA(III) can be separated from high-acid HLLW.

(1) $${\mathrm{RE}}^{3+}{+3\mathrm{NO}}_{3}^{-}{+3\mathrm{CMPO}/\mathrm{SiO}}_{2}{\mathrm{-P}}_{\left(\mathrm{resin}\right)}\;\Leftrightarrow \;{\mathrm{RE}(\mathrm{NO}}_{3}{)}_{3}\cdot {3\mathrm{CMPO}/\mathrm{SiO}}_{2}{\mathrm{-P}}_{\left(\mathrm{resin}\right)}$$

(2) $$M^{3+}{+3\mathrm{NO}}_{3\;}^{-}{+3\mathrm{TODGA}/\mathrm{SiO}}_{2}\mathrm{-P}\;\Leftrightarrow \;{M(\mathrm{NO}}_{3}{)}_{3}\cdot {3\mathrm{TODGA}/\mathrm{SiO}}_{2}\mathrm{-P}\;(M(\mathrm{III})=\mathrm{RE}(\mathrm{III})\;\mathrm{and}\;(\mathrm{An}))$$

The N-containing R-BTP and its derivatives R-BTBP and R-BTPhen have attracted wide attention due to their high selectivity for MA(III) from high-acid solutions since 1999 [50,51]. As they are composed of C, H, O, and N, they could be completely combustible after use up, which avoids secondary radioactive waste accumulation. The separation properties and stability of R-BTP strongly depend on the structure of alkyls. Several R-BTP/SiO_2_-*P* sorbents have been prepared, including Me-BTP/SiO_2_-*P*, Et-BTP/SiO_2_-*P*, nBu-BTP/SiO_2_-*P*, nHex-BTP/SiO_2_-*P*, C8-BTP/SiO_2_-*P*, isoBu-BTP/SiO_2_-*P*, isoHexyl-BTP/SiO_2_-*P*, isohexptyl-BTP/SiO_2_-*P*, cyhexptyl-BTP/SiO_2_-*P*, CA-BTP/SiO_2_-*P*, Me_2_-CA-BTP/SiO_2_-*P*, isoPentyl-BTBP/SiO_2_-*P*, CyMe_4_-BTPhen/SiO_2_-*P,* etc. The effects of the side alkyl chain length were studied. Among Me-BTP/SiO_2_-*P*, Et-BTP/SiO_2_-*P*, nBu-BTP/SiO_2_-*P*, nHex-BTP/SiO_2_-*P*, and n-C_8_H_17_-BTP/SiO_2_-*P*, nBu-BTP/SiO_2_-*P* exhibited the best adsorption toward Am(III), with a high separation factor(*SF*) about 100, while the longer side-chain ligands exhibited the lower leakage of R-BTP in high-acid solution. The ligands with branched side chains, such as isoBu-BTP/SiO_2_-*P* and isoHexyl-BTP/SiO_2_-*P*, have better acid stability than those with straight chains, e.g., nBu-BTP/SiO_2_-*P* and nHex-BTP/SiO_2_-*P*. At the same time, Barbette et al. [52] covalently combined tetraazamacrocyclic compounds substituted by N-tripropionic acid (or N-triacetic acid) onto silicon gel to synthesize a variety of modified mesoporous SiO_2_ molecules, which can reduce the amount of plutonium and americium. The experimental results show that after comprehensive purification of wastewater containing radioactive elements, the residual radioactivity in the wastewater is below the detection limit. Meyer et al. [53] synthesized a silicon-based hybrid material using sol–gel technology. The study found that the hybrid material containing the diaminoethyl group showed an efficient extraction ability for Am (III) at a concentration of less than 0.01 M nitric acid, while the extraction of Pu (IV) was conducted through anion exchange at a concentration of more than 3 M nitric acid. However, the material has low selectivity for americium and cannot effectively separate americium and europium. With the ring structure exhibiting good radiation stability due to the precluding of α-H, such as Me_2_-CA-BTP/SiO_2_-*P*, it was suggested to be the most promising extractant that can not only separate Am from Ln(III) in pH = 2-NaNO_3_ solution or 0.1 M HNO_3_ but can also directly separate Am from simulated 3 M HNO_3_ HLLW. Furthermore, the adsorption mechanism was also studied using EXAFS, FT-IR, and XPS, which proved to be Equation (3). Furthermore, Am(III) and Cm(III) are expected to exhibit similar adsorption behaviors, which has been proved in the case of nBu-BTP/SiO_2_-*P*.

(3) $${(M}^{3+}{+3\mathrm{NO}}_{3}^{-}+n\left(\mathrm{R-BTP}\right){=M(\mathrm{NO}}_{3}{)}_{3}{\left(\mathrm{R-BTP}\right)}_{n},\;n=1,\;2,\;3)$$

Based on the above adsorbents, this study proposes two MAREC (extraction chromatography for recovering trace actinide elements from high-level radioactive waste) processes [54]. One is a two-step process in which MA(III) and Ln(III) are co-separated from higher-acid HLLW using CMPO/SiO_2_-*P* or TODGA/SiO_2_-*P*, and the adsorbed MA(III) and Ln(III) can be desorbed by water. Then, after acidity or NO_3_^−^ concentration adjustment, MA(III) and Ln(III) can be mutual separated from each other using HDEHP/SiO_2_-*P* or Cyanex301/SiO_2_-*P* or R-BTP/SiO_2_-*P*. The other is a very challenging separation process where MA is directly separated from higher-acid HLLW with just one step, which might be realized using isohexyl-BTP/SiO_2_-*P* or Me_2_-CA-BTP/SiO_2_-*P*.

Moreover, SiO_2_-based SiPyR-N3, SiPyR-N4, and AR-01 macro-reticular resin containing N-methylbenzimidazole and N,N9-dimethylbenzimidazolium groups as exchange sites were prepared to adsorb anionic nitrato-complexes, mainly actinide species, which leads to actinide separation (U(IV), Np(IV), and Pu(IV)) from most other fission products, such as Cs(I), Sr(II), Mo(VI), Rh(III), Pd(II), and Tc(VII), as well as La(III) and MA(III). The corresponding flowsheet has also been proposed. Moreover, the adsorption kinetics was very fast, with Pu(IV) equilibrium obtained in 10 min using AR-01.

### 3.2. Lanthanide Separation

The separation of lanthanide and actinide elements is a crucial step in the nuclear fuel cycle and nuclear waste management, especially the separation of trivalent lanthanide elements from trivalent actinide elements [55,56]. Lanthanide elements, as neutron poisons, significantly affect the efficiency of actinide element transformation [57]. Through efficient lanthanum actinide separation technology, the transformation of actinide elements into low-toxicity, short-lived nuclides can be achieved, which is of great significance for reducing the long-term radiation toxicity of nuclear waste [58]. This work summarizes and organizes several ligands that are used for lanthanide element separation. The corresponding silicon-based hybrid adsorbent was prepared using the vacuum impregnation method. The summary and comparison of the adsorption and separation of lanthanide elements using silicon-based adsorbents are shown in Table 3.

HDEHP/SiO_2_-*P* has a good separation effect on trivalent lanthanide elements. The new HDEHP/SiO_2_-*P* silicon-based adsorbent can effectively separate Y^3+^ in aqueous solutions containing high concentrations of strontium [59]. The results confirmed that HDEHP/SiO_2_-*P* has higher selectivity for Y^3+^ than Sr^2+^ in different acidic media. When the Sr-Y molar ratio is 2 × 10^3^, the *SF*_Y/Sr_ value of the adsorbent reaches as high as 1.93 × 10^3^. In addition, the dynamic separation of trace amounts of Y^3+^ from concentrated Sr^2+^ aqueous solution was successfully verified through column experiments, with a recovery rate of 100% for Y^3+^. In addition, HDEHP/SiO_2_-*P* can achieve the separation of MA and Ln groups under low-acid conditions. The adsorption of MA (III) and Ln (III) by HDEHP/SiO_2_-*P* decreases with the increase in HNO_3_ concentration and shows preferential adsorption of Ln (III) over MA (III). Zhou et al. [60] developed a β-amino phosphonic acid resin based on silica/polymer, prepared using the vacuum impregnation method for separating lanthanide elements from chloride media. At pH = 2.0, the separation factors (*SF*) of HEHAEP/SiO_2_-*P* for lanthanide elements are as follows: Er/Ho is 2.35, Tm/Er is 3.62, Yb/Tm is 3.14, and Lu/Yb is 1.23, which are superior to other reported impregnating resins. And the adsorbed lanthanide elements can be completely eluted using 4.0 mol/L HNO_3_. The TRPO/SiO_2_-*P* silicon-based composite material prepared via vacuum impregnation using trialkylphosphine oxide (TRPO) as the functional ligand has excellent adsorption and separation effects on scandium. The study investigated the separation efficiency of 0.2 M H_2_ SO_4_ and 5 M HCl solutions. The results showed that TRPO/SiO_2_-*P* resin exhibited excellent separation performance in sulfuric acid and hydrochloric acid media, with separation factors (*SF*_Zr/Sc_) reaching 380 and 977, respectively. Using oxalic acid (H_2_C_2_O_4_) as the eluent, efficient elution of scandium and zirconium was achieved, with an elution efficiency close to 100%. Nitrogen containing silicon-based adsorbents such as Me_2_-CA-BTP/SiO_2_-*P* can achieve the separation of Ln (III) and Am (III) in pH = 2 NaNO_3_ solution or 0.1 M HNO_3_ and can directly separate Am from simulated 3 M HNO_3_ high-level radioactive liquid waste. A macroporous silicon-based polymer resin (TODGA/SiO_2_-*P*) was modified with TODGA (trioctyldiglycolamine) for the separation of zirconium and scandium from nitric acid medium. This resin can be prepared using the vacuum impregnation method and has good mechanical stability and fast kinetic properties. The adsorption rate of TODGA/SiO_2_-*P* resin for zirconium is close to 100%, and it effectively separates scandium in 1 M HNO_3_ solution with a separation factor (*SF*_Zr/Sc_) of up to 3694. This study provides a new method for the efficient separation of zirconium and scandium from complex solutions.

### 3.3. Sr and Cs Separation

As the representative fission product, ^90^Sr has a half-life of 28.79 years and a yield of 5.89% during ^235^U fission [61,62]. Similarly, ^137^Cs has a half-life of 30.17 years, a high amount of Cs group of ~3.6 kg/t HU, and 45 GWd/t [63] and contributes more than 80% of the heat and radioactivity of HLLW in the first few hundred years [64,65]. Both Sr and Cs pose serious potential radiation hazards to the environment and humans. The high content of ^89^Sr and ^137^Cs as a heat generator is not conducive to the vitrification of HLLW and may decrease its stability, thus leading to radioactive leaking into the environment [66,67]. In addition, ^89^Sr and ^137^Cs are sources of β emitters and can be used as energy generators. Furthermore, ^89^Sr and Ca have similar chemical properties. They are absorbed through the gastrointestinal tract and easily accumulate in the body to become part of the bone marrow tissue and destroy hematopoietic cells, as they are radioactive [68,69,70]. Moreover, the daughter nuclide of ^90^Sr, namely ^90^Y, has a half-life of 64 h and emits pure β-radiation, with an average energy of 0.9 MeV. These properties make it an ideal radionuclide for medical treatments, thereby attracting increasing attention in the field. Therefore, separating and recovering Sr and Cs from radioactive waste is of great significance.

#### 3.3.1. Sr Separation

Nowadays, studies on the separation and recovery of radioactive ^90^Sr mainly focus on the following aspects: i.e., highly acidic HLLW, accident wastewaters such as waste seawater generated in the Fukushima accident, and low-level liquid waste in the daily operation of power plants. Except selectivity, different systems have differential requirements for separation materials. High stability is typically required for the materials during Sr(II) extraction from highly acidic HLLW, while for that in seawater system, high selective sorption ability toward Sr(II) in the existence of large amounts of competitive ions (such as Na^+^, K^+^, Ca^2+^, and Mg^2+^) would be mostly needed [71,72].

Up to now, materials that can effectively extract Sr(II) from higher-acid HLLW are very rare, such as crown ether extractants (e.g., 4′,4′(5′′)-di(tert-butylcyclohexano)-18-crown-6 (DtBuCH18C6) and dicyclohexano-18-crown-6 (DCH18C6)) and amide pod ether extractants (e.g., TODGA). Among of them, DtBuCH18C6 is the most recognized one due to its size match with Sr(II) and is used in the SREX method developed by Argonne National Laboratory, but it suffered from the problems of third phase, which may be caused by the relatively high solubility of DtBuCH18C6 in the aqueous phase and the lower solubility of the Sr-DtBuCH18C6 complex in the organic phase [73]. To overcome the shortcomings, a Sr resin was elaborately developed by impregnating 1 M DtBuCH18C6 in n-octanol into the pores of XAD-7, but it was not further considered due to its inconspicuous low adsorption ability. A DtBuCH18C6/SiO_2_-*P* sorbent was prepared using the vacuum impregnation method. It exhibited good selectivity toward Sr in 1–3 M HNO_3_ solution, with the adsorption capacity 104.6 mg Sr/g and *K*_d_ following the order of Sr^2+^ >> Ba^2+^ >> K^+^, Cs^+^, La^3+^, Y^3+^ (the adsorption mechanism is shown in Equation (4)). Sr and Ba are adjacent elements of the same alkali earth metal group and share some similar properties, which leads to their co-sorption by DtBuCH18C6/SiO_2_-*P*. The other drawback is the high leakage of DtBuCH18C6 into the aqueous environment, with a TOC value as high as 522.3 mg/L due to the six oxygens in it, leading to good affinity with water. To overcome the problem, a series of modified sorbents were prepared by adding various synergistic extractants, such as TBP, 1-dodecane, and n-octanol [74], to form hydrogen bonds to increase its hydrophobicity, and their selective adsorption toward Sr(II) were also intensively evaluated and compared (Table 3). The distribution coefficients (*K*_d_) of Sr(II) in 2 M HNO_3_ obeyed the following order: DtBuCH18C6/SiO_2_-*P* > (DtBuCH18C6+Octnol)/SiO_2_-*P* > (DtBuCH18C6+Dodecanol)/SiO_2_-*P* > (DtBuCH18C6+TBP)/SiO_2_-*P*. Additionally, the DtBuCH18C6 leakage follows the order of DtBuCH18C6/SiO_2_-*P* > (DtBuCH18C6+TBP)/SiO_2_-*P* > (DtBuCH18C6+Dodecanol)/SiO_2_-*P* > (DtBuCH18C6+Octnol)/SiO_2_-*P*. In a word, after modification, the leakage of DtBuCH18C6 was inhibited, while some other effects occurred, such as increased selectivity, increased adsorption capacity, etc. Another sorbent, TODGA/SiO_2_-*P,* also exhibited satisfactory selectivity toward Sr in 0.5–4 M HNO_3_ solution, with *K*_d_ following the order Sr^2+^ >> Ba^2+^ >> Na^+^, K^+^, Cs^+^, Rb^+^, Ba^2+^, Ru^3+^. Based on such a sorbent, Sr(II) was successfully separated from Na(I), K(I), Cs(I), Rb(I), Ba(II), and Ru(III) in 2.0 M HNO_3_ solution. Compared with the DtBuCH18C6 sorbent, the leakage of TODGA/SiO_2_-*P* due to the dissolution of the extractant is much lower, while its selectivity is mediocre. A novel CEPA@SBA-15-APTES adsorbent was obtained via amino modification and phosphorylation of SBA-15. CEPA@SBA-15-APTES and HEMAP/SiO_2_-*P* exhibit rapid kinetics in the presence of 3 M HNO_3_, reaching adsorption equilibrium in 5 min and 1 min, with *K*_d_ following the order Sr^2+^ >> Ba^2+^, K^+^, Cs^+^, Na^+^, Pd^2+^, Ru^3+^, Y^3+^, Mo^4+^, La^3+^/Sr^2+^>Y^3+^ >> Nd^3+^ > Mo^4+^, La^3+^, Ru^3+^, Dy^3+^. In summary, due to the similarity between Sr and Ba, the presence of Ba is not conducive to the separation of Sr. At present, there are few reports on the selective extraction of strontium ligands from high-acid solutions. A summary and comparison are given in Table 3.

(4) $$({\mathrm{nSr}}_{2(\mathrm{aq})}^{2+}{+2\mathrm{nNO}}_{3(\mathrm{aq})}^{-}+\mathrm{mDtBuCH}18C6/{\mathrm{SiO}}_{2}\mathrm{-P}\;\Leftrightarrow \;{\mathrm{nSr}(\mathrm{NO}}_{3}{)}_{3}\cdot \mathrm{mDtBuCH}18C6/{\mathrm{SiO}}_{2}\mathrm{-P})$$

On the other hand, various materials have been prepared to selectively extract Sr(II) from a low-acidic solution, including organic and inorganic, as well as natural and artificial, such as zeolite, metal sulfide, titanate, titanium silicate, and organic functional sorbents. Due to the unsuitability of nano-sized and irregularly shaped materials for dynamic column separation operations, it is of great significance to make them have regular shapes and be suitable for continuous column separation. As such, both organic and inorganic materials have been used to combine SiO_2_-*P* or SiO_2_ to prepare sorbents. Different titanate-based inorganic silica adsorbents were prepared using the sol–gel method and in situ growth combined with vacuum impregnation, as summarized in Table 4. K_2_Ti_6_O_13_/SiO_2_ showed good adsorption toward Sr in pH 3–7 solution, with the adsorption amount being about 15 mg Sr/g and equilibrium obtained over 8 h. Hot-column experiments proved that K_2_Ti_6_O_13_/SiO_2_ can treat about an 80 bed volume of contaminated simulated seawater. Na_2_TinO_2n+1_/SiO_2_ exhibited good adsorption toward Sr(II) in a wide pH range of 3–10, with the adsorption capacity as high as 66.37 mg Sr/g, the adsorption equilibrium obtained within 10 min, and the *K*_d_ following the order Sr^2+^ > Ba^2+^ >> Mg^2+^, Ca^2+^, Cs^+^, K^+^. Moreover, it can treat a 950 bed volume of simulated waste seawater at the rate of 30 Bed volume per hour via the column experiment with almost no Sr(II) leakage, which seems to be very effective. The h-WO_3_/SiO_2_ adsorbent was prepared using the hydrothermal method, which showed excellent selectivity toward Sr^2+^ in pH = 4 solution, with a *K*_d_ of more than 2000 cm^3^/g in the presence of Ca^2+^, Mg^2+^, La^3+^, and Dy^3+^ and equilibrium obtained within 15 min. The ZrP/MSP material prepared via the liquid-phase grafting method has good adsorption effect on Sr(II) in pH 4–7 solution, with a maximum adsorption capacity of 100.77 mg Sr/g and an equilibrium time of 1.5 h. Meanwhile, ZrP/MSP still achieved a 92.5% removal rate of Sr(II) in the presence of excess Na^+^, K^+^, Ca^2+^, Mg^2+^, Ba^2+^, and Zn^2+^. Sb_2_O_5_/SiO_2_ has good adsorption selectivity for Sr(II) in pH = 6–9, with adsorption equilibrium reached in 5 min at pH = 6 and an adsorption capacity of 160.6 mg Sr/g. In addition, the results of the dynamic column experiment showed that the device can efficiently process a simulated seawater solution with a volume of 268 bed, without any leakage of Sr(II) during the process. In the separation of Sr from a low-acid medium or seawater using inorganic materials, most of the investigations focus on the selective adsorption of Sr followed by solidification, while reusability is not of concern yet. By using methylacrylic acid as a functional monomer, ethylene glycol dimethacrylate as a cross-linking agent, and SiO_2_ as a support, a resin named SiMaC was prepared via an in situ polymerization method. The as-prepared SiMaC adsorbed Sr(II), increasing with the pH in the range of 2–10, with the adsorption capacity as high as 142.5 mg Sr/g, and kept the uptake rates of Sr(II) in river water, lake water, and seawater at 99%, 99%, and 76%, respectively. More importantly, it can still be reused, which is a great improvement compared with these inorganic materials.

Moreover, studies on silica-based sorbents for the separation of Sr and Y for the preparation of radiopharmaceutical waste were also carried out. TODGA/SiO_2_-*P* exhibited adsorption toward both Sr^2+^ and Y^3+^ in 3 M HNO_3_ solution, and the adsorbed Sr^2+^ and Y^3+^ were successively desorbed using H_2_O and 0.01 M DTPA [75]. HDEHP/SiO_2_-*P,* (HDEHP+dodec)/SiO_2_-*P*, and (HDEHP+Hexa)/SiO_2_-*P* also exhibited better adsorption toward Y^3+^ compared with Sr^2+^ in both 0.001–0.5 M HNO_3_ and HCl solution. Sr^2+^ and Y^3+^ were separated from each other from 0.5 M HCl and HNO_3_ solution by successively desorbing with 0.5 M HCl (HNO_3_) and 3 M HCl (HNO_3_) [76,77,78]. Also, the separation and recovery of Y(III) from a Sr^2+^-Y^3+^ mixture were realized using CMPO/SiO_2_-*P* and (CMPO+Dodec)/SiO_2_-*P* [79]. Although Sr^2+^ and Y^3+^ separation was achieved, it is difficult to meet the requirements of a strontium-adsorbed Sr-Y generator, as Sr^2+^ was poorly adsorbed compared with Y^3+^. Therefore, it still requires sorbents with strong and good adsorption toward Sr, and ^90^Y produced by decay can be desorbed according to actual needs.

#### 3.3.2. Cs Separation

The materials that can effectively separate Cs include organic materials (mainly macrocyclic crown-calixarene) and inorganic materials (such as ammonium phosphomolybdate (AMP), zeolite, ferrocyanide, titanium silicate, etc.) [80]. Macrocyclic crown-calixarene with different structures and AMP have been combined with SiO_2_-*P* or SiO_2_ to make sorbents suitable for column operation. The high selectivity of macrocyclic crown-calixarene toward Sr(II) is due to the size matching between the calixarene cavity and metal ion, as well as Π-bonding interactions with the arene groups and structural reorganization of the molecule [81]. 1,3-[(2,4-diethyl-heptylethoxy)oxy]-2,4-crown-6-calix[4]arene (Calix[4]arene-R14) is the most studied one. Nevertheless, due to its large molecular size, with the molecular weight close to 1000, macrocyclic crown-calixarene has poor affinity with organic compounds, so it is modified by adding a hydrophilic modifier during the preparation of SiO_2_-*P*-based sorbents, such as TBP [82], methyloctyl-2-di-methy-lbutanemide (MODB), dodecanol [83], and dodecanol+DBS [84]. The as-prepared (Calix[4]R14+TBP)/SiO_2_-*P* [85], (Calix[4]+MODB)/SiO_2_-*P*, (Calix[4]+dodecanol)/SiO_2_-*P*, and (Calix[4]+dodecanol+DBS)/SiO_2_-*P* all exhibited high selectivity toward Sr in high-concentration HNO_3_ solution, and the modifiers affected the crown-calixarene performance, such as the best adsorption selectivity acidity and equilibrium time. For comparison, the adsorption properties of crown-calixarene-based materials toward other metal ions are shown in Table 5. For example, the adsorption of (Calix[4]+dodecanol+DBS)/SiO_2_-*P* toward Sr decreased with HNO_3_ concentration, but it still maintained high adsorption ability in 3 M HNO_3_ solution, which is different from (Calix[4]+dodecanol)/SiO_2_-*P,* which has good adsorption toward Sr in 2.0 M HNO_3_. The reason is due to ion exchange caused by the low acidity of DBS (dodecyl benzenesulfonic acid). Moreover, new macrocyclic crown-calixarenes were designed and also made into sorbents, such as BnOCalix[4]C6/SiO_2_-*P* [86] and (CalixBNaphC)@SiO_2_-*P* [87], both of which showed good selectivity toward Sr in 3 M HNO_3_ solution. The adsorption mechanism is summarized in Equation (5). A new process entitled PCEC (Partitioning of Cesium by Extraction Chromatography) for the individual separation of Cs(I) based on the above-mentioned sorbents was proposed and proved, by which Cs can be successfully separated from other fission products. Moreover, the hybrid macrocyclic compound (Calix[4]+DtBuCH18C6)/SiO_2_-*P* was prepared, by which Sr and Cs were efficiently and simultaneously captured from 3 M HNO_3_ solution and eluted using water, and the leakage of DtBuCH18C6 was inhibited in the case of (Calix[4]+DtBuCH18C6)/SiO_2_-*P* after the higher-content non-polar Calix[4] added. Also, the inorganic sorbent AMP/SiO_2_ was prepared, whose affinity toward Cs decreased with the HNO_3_ concentration. Nevertheless, it still achieved a *K*_d_ over 300 in 3.0 M HNO_3_, reaching adsorption equilibrium within 30 min and achieving good γ irradiation stability. Additionally, its solidification of Cs following adsorption was studied, which achieved better results after modification, but its selectivity has not been studied yet.

(5) $${\mathrm{Cs}}^{+}{+\mathrm{NO}}_{3}^{-}+\mathrm{Calix}\left[4\right]{\mathrm{arene-R}14/\mathrm{SiO}}_{2}\mathrm{-P}\;\Leftrightarrow \;\;[\mathrm{CsC}\mathrm{alix}\left[4\right]{\mathrm{arene-R}14/\mathrm{SiO}}_{2}{\mathrm{-P}]\mathrm{NO}}_{3}$$

### 3.4. Separation of Platinum Group Metal Fission Products

The fission products of platinum group metals (PGMs) primarily include Ru, Rh, and Pd [88,89]. The total content of platinum group metals in spent fuel is relatively high and has certain economic recovery value. It is estimated that each ton of spent fuel in a reactor with a burnup of 33 GWd/t contains approximately 1 kg of palladium [90]. It is worth noting that their content is expected to be higher in fast reactors. PGMs can not only be used for high-end industrial applications such as catalysts and permanent magnets but can also reduce the volume of radioactive waste through separation and reuse [91]. Moreover, PGMs in HLLW have serious adverse effects on the subsequent solidification process, so it would be necessary to separate PGMs from HLLW, which has not received enough attention yet.

Several kinds of sorbents based on SiO_2_ and SiO_2_-*P* have been prepared for PGM separation, most of which are N-containing, where the N atom acts as a Lewis base donating the electron pair and as a hard donor according to the hard–soft acid–base theory. For clarification, these sorbents are summarized and compared in Table 6. Sorbents based on the MOTDGA (N,N″-dimethyl-N,N″-di-n-octyl-thiodiglycolamide) series, Crea (N′,N′-di-n-hexyl-thiodiglycolamide), and DAMIA-EH (2,2′-[(2-ethyl-hexyl)imino]bis[N,N-bis(2ethylhexyl)acetamide]) sharing similar structures containing O, S, or N coordination sites, after modification by TOA (Tri-n-octylamine) or dodecanol, all exhibited excellent sorption toward Pd with fast kinetics, but few have good adsorption toward Ru, especially Rh. Ye et al. grafted thiourea groups onto SiO_2_-*P* to prepare a SiPS-TU adsorbent containing a S ligand for the adsorption and separation of platinum group metals. In addition, dNbpy (g 4,4′-dinonyl-2,2′-dipyridyl) also contains a N ligand that can efficiently separate Pd from HLLW and has fast kinetics. The newly developed N-containing materials SiVpC/SiO_2_ and 2AT-SiAaC (in the past two years) can achieve efficient enrichment and recovery of Pd in simulated high-level radioactive waste. Among them, 2AT-SiAaC has good affinity selectivity for Pd (*K*_d_ (distribution coefficient) ≥ 10,344.2 mL/g; *SF*_Pd/M_ (separation factor) ≥ 613.7). The dynamic column experiment results show that 2AT-SiAaC has a good separation effect on Pd in simulated HLLW, with an enrichment coefficient (C/C_0_) of about 14 and a recovery rate of nearly 99.9%. In particular, the amine-functionalized mesoporous silica (SBA-15-TEPA) adsorbent constructed from mesoporous silica also exhibits excellent selectivity (separation factor > 5565) toward Pd over a wide range of acidities. The modifier exhibited a certain effect on the performance of the sorbents, as shown in Table 7. Furthermore, increasing the temperature is helpful for the adsorption of Ru, which has been found in the cases of (Crea + TOA)/SiO_2_-*P* and (MOTDGA-TOA)/SiO_2_-*P*, but a relatively long contact time is needed to reach adsorption equilibrium.

The specially designed R-BTP for MA(III) separation also exhibited good adsorption toward Pd, such as Me_2_-CA-BTP/SiO_2_-*P*, isoHex-BTP/SiO_2_-*P*, and isoBu-BTP/SiO_2_-*P*, by which Pd was separated from other fission products during the separation of MA by column experiments. Furthermore, isoBu-BTP/SiO_2_-*P* exhibited good adsorption toward Ru, Rh, and Pd, with uptake rates over 90% in nitrate solution at 328 K, suggesting it to be a very promising material in PGM fission product separation. As a result, a process based on isoBu-BTP/SiO_2_-*P* to co-separate Am, Ru, Rh, and Pd has been proposed.

Moreover, an inorganic sorbent hexacyanoferrates KNiHC/SiO_2_ was prepared to separate Pd from 3 M HNO_3_ solution via ion exchange and the oxidation–reduction mechanism, which is very rare, as before this, most are organic materials. In a word, the selective separation of Pd from HLLW is easy to achieve by N- or S-containing ligands, but the separation of Ru, especially for Rh are difficult. Additionally, it has been widely proved that increasing the temperature is beneficial to the adsorption of Ru, calling for that the effect of temperature needs to be specially considered in the separation of Ru in future works.

### 3.5. I Separation

The Fukushima Daiichi nuclear power plant accident in Japan resulted in a large amount of radioactive contaminated wastewater. Reactor cooling water (RCW) is collected following contact with damaged nuclear fuel debris [92,93]. As a result, the primary radioactive nuclides present are soluble cesium isotopes (^134^Cs, ^137^Cs), strontium (^90^Sr), and iodine (^131^I, ^129^I) [94]. The half-life of ^129^I is 1.7 × 10^7^ years, which is the main long-term risk driving factor for shallow land disposal facilities [95]. Radioactive iodine is a major fission product that is harmful to the human body. If ingested and enriched in the thyroid gland, it may lead to radiation damage [96,97]. Meanwhile, untreated iodine pollutants released into the environment can contaminate soil, air, and groundwater, causing long-term radiation ecological risks [98,99]. Consequently, the separation and removal of radioactive iodine hold immense significance.

Adsorption materials that are effective in the adsorption and separation of iodine ions include silver, hydrotalcite, SiPyR-N4, etc. A new silicon-based composite adsorbent was prepared for the removal of iodine ions via vacuum impregnation and in situ polymerization/crystallization. The silver complex of thiourea (Ag(tu)_3_NO_3_) was grafted onto a silica-based copolymer carrier (SiO_2_-*P*) to prepare a macroporous silica-based silver adsorbent [100]. The adsorbent has good thermal stability below 200 °C and exhibits fast adsorption kinetics for iodide ions in both pure water and 0.6 M NaCl solution, with an equilibrium time of 10 min. Mg-Al hydrotalcite was introduced into porous silica through in situ precipitation crystallization to prepare a Mg-Al-LDO/SiO_2_ adsorbent [101]. When the adsorbent dosage is 0.05 g/100 mL in a 30 mg/L iodine ion solution, the removal efficiency of iodine ions reaches 99.81% within 5 min. This study demonstrates the high efficiency and practicality of Mg-Al-LDO/SiO_2_ composite materials in treating iodine ion wastewater, providing a new solution for nuclear waste treatment and environmental protection. To improve the removal efficiency and adsorption capacity of iodine ions, a quaternized silicon-based ion-exchange resin was prepared. Efficient removal is achieved through ion-exchange reactions between surface quaternary ammonium groups (such as SiPyR-N4) and iodide ions [102]. At pH 6.0, in a solution containing 0.1 mmol/L iodine ions, the removal rate can reach over 96% within 30 min. The maximum adsorption capacity for iodine ions can reach 148.23 mg/g. SiPyR-N4 exhibits excellent fixation ability for iodine ions at a flow rate of 8 mL/min, with a maximum dynamic capacity of 124 mg/g, while the commercial products D201 and IRA-900 immediately leak under the same conditions. Regeneration experiments have shown that SiPyR-N4 can be reused. Riley et al. [103] successfully prepared a silver-loaded aerogel by immersing an aluminosilicate aerogel into silver nitrate solution. Its iodine adsorption capacity is four times that of traditional silver zeolite. In summary, this work proposes a promising adsorbent for capturing and enriching iodine from wastewater on a large scale.

## 4. Conclusions

This review reports on the research progress of macroporous silicon-based adsorbents in separating key nuclides (including ammonium chloride, strontium, and cesium, which are platinum group metal fission products) in the nuclear fuel cycle. For the separation of minor actinides, many kinds of sorbents have been developed that universally exhibit selectivity under certain conditions. Both CMPO/SiO_2_-*P* and TODGA/SiO_2_-*P* can co-adsorb MA and Ln in high-acid solution, while Cyanex301/SiO_2_-*P* and HDEHP/SiO_2_-*P* can only selectively adsorb MA in mild-acidic solution. Although its stability is widely controversial, R-BTPs/SiO_2_-*P* would be the most attractive sorbents that can selective separate MA from high-acid solution, and a very challenging one-step MAREC process has been typically proposed based on such a sorbent. Regarding the separation of Sr and Cs from high-acid solution, very limited materials have been developed, and they suffered from some drawbacks, such as a high leakage rate in the liquid phase, suggesting that further development is needed. For the fission products of PGMs, Pd would be selectively adsorbed in HNO_3_ system and desorbed by thiourea easily. Additionally, increasing the temperature could promote the adsorption of Ru, while few materials are found to be effective for the adsorption of Rh. In summary, macroporous silicon-based adsorbents have excellent effects on the separation of key radioactive nuclides in the nuclear fuel cycle. However, the silicon-based resin prepared via the vacuum impregnation method still has some drawbacks, such as a certain leakage rate and low adsorption capacity. In addition, macroporous silicon-based resins generally have lower mechanical strength. Therefore, optimizing the preparation process of macroporous composite adsorbents, reducing the leakage rates, and improving the adsorption capacity and mechanical strength of adsorbents are still directions that we need to continue exploring.

## Figures and Tables

**Table 1:** Various extractants or ligands were previously used in the historical literature.

Name of Adsor.	Full Name	Chemical Structure	Target Element
HDEHP	Di(2-ethylhexyl)phosphoric acid	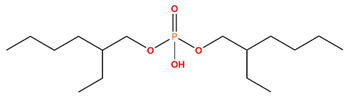	Gd, Eu, Ce, Am, Sr, and Y
Cyanex301	Bis(2,4,4-trimethylpentyl) dithiophosphinic acid	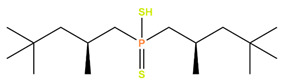	Gd, Eu, Ce, and Am
CMPO	Octyl(phenyl)-N, N-diisobutyl-carbamoylmethylphosphine oxide	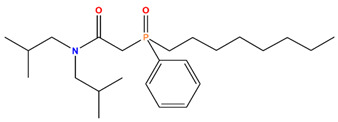	Mo, MA, Cs, Sr, and Ru
TODGA	N,N,N′,N′-tetraoctyl-3-oxapentane-1,5-diamide	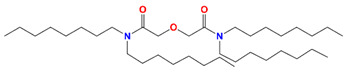	Am, Nd, Sm, Y, and Sc
R-BTP	2,6-bis-(5,6-dialkyl-l,2,4-triazine-3-yl)-pyridine	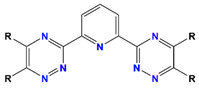	Am and Dy
Isobutyl-BTP	(2,6-di(5,6-diisobutyl-1,2,4-triazin-3-yl) pyridine	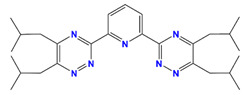	Am, Pu, and Dy
Isohexyl-BTP	2,6-bis(5,6-dii-sohexyl)-1,2,4-triazin-3-yl) pyridine	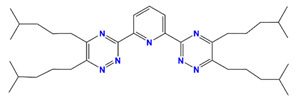	Am, Pu, Dy, and Eu
Me_2_-CA-BTP	2,6-bis(5,6,7,8-tetrahydro-5,8,9,9-tetramethyl-5,8-methano-1,2,4-benzotriazin-3-yl) pyridine	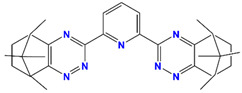	Am, Dy, Gd, and Eu
CA-BTP/SiO_2_-*P*	bis-2,6-(5,6,7,8-tetrahydro-5,9,9-trimethyl-5,8-methano-1,2,4-benzotriazin-3-yl) pyridine	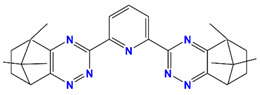	Am and Pu
AR-01	N-methylbenzimidazole and N, N9-dimethylbenzimidazolium groups as exchange sites	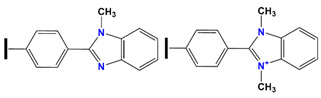	U, Np, and Pu
Calix[4]arene-R14	1,3-[(2,4-diethyl-heptylethoxy) oxy]-2,4-crown-6-calix[4]arene	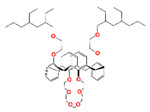	Cs, Rb, K, Na, and Sr
DtBuCH18C6	4,4′,(5′)-di-(tert-butylcyclohexano)-18-crown-6	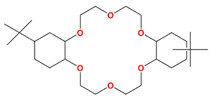	Sr, Ba, K, Cs, La, and Y
MOTDGA	N,N′-dimethyl-N,N′-di-n-octyl-thiodiglycolamide	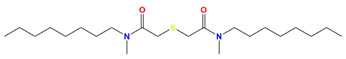	Pd, Zr, Mo, Ru, and Rh
Crea	N′,N′-di-n-hexyl-thiodiglycolamide	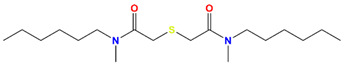	Ru, Rh, and Pd
DAMIA-EH	2,2′-[(2-ethyl-hexyl) imino]bis[N,Nbis(2ethylhexyl)acetamide]	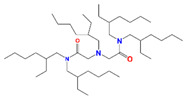	Pd and Ru
TOA	Tri-n-octylamine	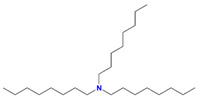	Pd
DTPA	diethylenetriaminepentaacetic acid	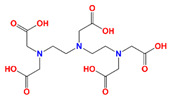	Sr
SiO_2_-*P*	P: styrene-divinylbenzenecopolymer	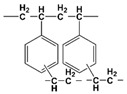	/

**Table 2:** Summary and comparison of silicon-based sorbents on adsorption and separation of actinides.

Sorbent	Equilibrium Time	*SF*	Good (Best) Adsorption Acidity	*K*_d_ Order	Column Results	Leakage Rate
HDEHP/SiO_2_-*P*	0.5 h	/	0.1–0.2 M	Gd(III) > Eu(III) > Ce(III) > Am(III)	/	<1%
Cyanex301/SiO_2_-*P*	1 h	*SF*_Am/Eu_: 500–5000	pH 4	Am(III) >> Gd(III), Eu(III), Ce(III)	Am(III) was separated from Gd(III), Eu(III), and Ce(III).	/
CMPO/SiO_2_-*P*	0.5–1 h	/	/	Zr(IV) > Mo(VI) > RE(III) ~ MA(III) > Pd(II) > Cs(I), Sr(II), Ru(III)	MA(III) and Ln(III) are expected co-separated.	0.4−0.8%
TODGA/SiO_2_-*P*	0.5 h	/	/	/	Am(III), Nd(III), Sm(III), and Y(III) were separated from Rh(III), Mo(IV), Sr(II), Ce(III), and Pr(III).	<0.02%
nBu-BTP/SiO_2_-*P*	1 h		Increase with NaNO_3_ concentration	Am(III), Cm(III) >> Gd(III), Eu(III) >> Ce(III), Cs(I), Sr(II), Zr(IV), Mo(IV), Ru(III), Rh(II)	Am(III) was separated from Y(III), Ce(III), Eu(III), and Gd(III).	
isoBu-BTP/SiO_2_-*P*	3 h for stable elements and 0.5 h for trace amount of Am	*SF*_Am/Ln_(Ln = Ce, Nd, Gd, Eu) about 100	pH = 1 + 1–4 M NaNO_3_	Am(III) >> Dy(III) > Gd(III) > Nd(III), Ce(III)	Am was separated from Dy and the other light Ln(III).	0.15% in 0.01 M HNO_3_ and 1.2% under γ irradiation for 5 months in 0.01 M HNO_3_
isoHexyl-BTP/SiO_2_-*P*	15–24 h	*SF*_Am/_Eu > 100	2–4 M HNO_3_	Am, Pu >> Dy, Pd > Gd, Eu >> La, Ce, Nd, Sm, Sr, Y, Zr, Mo, Tc, Ru in 3 M HNO_3_	Am was separated from Cs, Sr, Y, and Ce in 3 M HNO_3_.	0.1–4 M HNO_3_: TOC < 35 ppm
Me_2_-CA-BTP/ SiO_2_-*P*	12 h	*SF*_Am/Eu_ about 100 in 0.1–4 M HNO_3_	0.1–4 M HNO_3_ or 1–3 M NaNO_3_	Am > Dy > Gd, Eu > Pd, Sm, Mo, Zr, Cs, La, Ce, Y, Nd in 1 M HNO_3_	Am was directly separated from Sr, Y, Zr, Mo, Ru, Pd, and Ln in 3 M HNO_3_.	Stable in 1–3 M HNO_3_ under γ irradiation
CA-BTP/SiO_2_–*P*	>24 h	no adsorption toward Eu	0.5 M HNO_3_	Am > Pu, Zr, Tc, Ru > Ln(III), Sr, Y, Cs	/	Stable in dry state under γ irradiation
CyMe_4_-BTPhen/SiO_2_-*P*	0.5 h for trace amount of Am	*SF*_Am/Eu_ = 88.6 ± 0.1	4 M HNO_3_	Am > Pu > Pd, Mo, Ru > Zr, Tc, Y, Cs, Sr	/	0.1–4 M HNO_3_: TOC < 75 ppm
AR-01	10 min	/	6 M HNO_3_	U(IV), Np(IV), Pu(IV) >> FP in 6 M HNO_3_	U(IV), Np(IV), and Pu(IV) were separated from most other fission products.	/

“/” represents no research. *K*_d_ refers to the distribution coefficient.

**Table 3:** Summary and comparison of silicon-based sorbents on adsorption and separation of lanthanide.

Sorbent	Equilibrium Time	*SF*	Good (Best) Adsorption Acidity	*K*_d_ Order	Column Results	Stability
HDEHP/SiO_2_-*P*	5 h	*SF*_Sr/Y_: 1.93 × 10^3^	0.1 M	Y(III) > Sr(II)	Y(III) was separated from Sr(II)	<1%
HDEHP/SiO_2_-*P*	0.5 h	/	0.1–0.2 M	Gd(III) >Eu(III) > Ce(III) > Am(III)	/	<1%
HEHAEP/SiO_2_-*P*	12 h	*SF*_Er/Ho, Tm/Er, Yb/Tm, Lu/Yb_: 2.35, 3.62, 3.14, 1.23	pH = 2.0	/	Tm(III) was separated from Lu(III), La(III), and Am(III)	<0.1%
TODGA/SiO_2_-*P*	3 h	*SF*_Zr/Sc_: 3694	1 M HNO_3_	/	Am(III), Y(III), and Sc(III) were separated from Zr(II)	<0.02%
TRPO/SiO_2_-*P*	1 h for Sc	*SF*_Zr/Sc_: 380 and 977	0.2 M H_2_ SO_4_ and 5 M HCl	/	Sc(III) was separated from Zr(II)	/
Me_2_-CA-BTP/SiO_2_-*P*	12 h	*SF*_Am/Eu_ about 100 in 0.1–4 M HNO_3_	0.1–4 M HNO_3_ or 1–3 M NaNO_3_	Am > Dy > Gd, Eu > Pd, Sm, Mo, Zr, Cs, La, Ce, Y, Nd in 1 M HNO_3_	Am was directly separated from Sr, Y, Zr, Mo, Ru, Pd, and Ln in 3 M HNO_3_	Stable in 1–3 M HNO_3_ under γ irradiation

“/” represents no research.

**Table 4:** Summary and comparison of silica-based sorbents on Sr(II) adsorption in highly nitric solutions.

Sorbent	*K*_d_ Order	Capacity mg Sr(II)/g	Equilibrium Time	Good (Best) Adsorption Acidity	Column Results	Stability (TOC)	Sr(II) Desorber
DtBuCH18C6/Si-polymer	Sr^2+^ >> Ba^2+^ >> K^+^, Cs^+^, La^3+^, Y^3+^	104.6	>5 h	1–3 (2) M HNO_3_	/	424.8–634.6 ppm	/
(DtBuCH18C6+TBP)/SiO_2_-*P*	Sr^2+^ >> Ba^2+^ >> K^+^, Cs^+^, Na^+^, Pd^2+^, Ru^3+^, Y^3+^, Mo^4+^, La^3+^	/	About 2 h	0.5–5 (2) M HNO_3_	Sr(II) was separated from 2 M HNO_3_	251.2–352.7 ppm	water
DtDo/SiO_2_-*P* or (DtBuCH18C6+Dodec)/SiO_2_-*P*	Sr^2+^ >> Ba^2+^ >> K^+^, Cs^+^, Na^+^, Pd^2+^, Ru^3+^, Y^3+^, Mo^4+^, La^3+^	27–32	>5 h	1–5 (2) M HNO_3_	/	165.1–222.8 ppm	water
(DtBuCH18C6+Oct)/ SiO_2_-*P*	Sr^2+^ >> Ba^2+^ >> K^+^, Cs^+^, Na^+^, Pd^2+^, Ru^3+^, Y^3+^, Mo^4+^, La^3+^	/	About 60 min	/	Sr(II) was separated from 2 M HNO_3_	41 ppm	water
TODGA/SiO_2_-*P*	Sr^2+^ >> Ba^2+^ >> Na^+^, K^+^, Cs^+^, Rb^+^, Ba^2+^, Ru^3+^	/	About 10 min	0.5–4 (2) M HNO_3_	Sr(II) was separated from 2 M HNO_3_	TOC: 40 pm, 0.25%	water
(DtBuCH18C6+DBS+dodec)/SiO_2_-*P*	Sr^2+^ > Ba^2+^ > Zr > Na^+^ > Re^4+^ > Pd^2+^ > Mo^4+^, Ru^3+^, Nd^3+^, Dy^3+^	/	5 h	2 M HNO_3_	Sr(II) was separated from 3 M HNO_3_	/	Na-DTPA
(DtBuCH18C6+Dodec)/SiAaC-g-ABSA	Sr^2+^ > Ba^2+^ > Y^3+^ > Pd^2+^ > Ru^3+^ > Nd^3+^ > Mo^4+^, La^3+^	36.9	1 h	2 M HNO_3_	Sr(II) was separated from 2 M HNO_3_	TOC: 0.56%	/
CEPA@SBA-15-APTES	Sr^2+^ >> Ba^2+^, K^+^, Cs^+^, Na^+^, Pd^2+^, Ru^3+^, Y^3+^, Mo^4+^, La^3+^	112.6	5 min	3(4) M HNO_3_	Sr(II) was separated from 4 M HNO_3_	/	/
HEMAP/SiO_2_-*P*	Sr^2+^>Y^3+^ >> Nd^3+^> Mo^4+^, La^3+^, Ru^3+^, Dy^3+^	61.2	1 min	3 M HNO_3_	Sr(II) was separated from 3 M HNO_3_	/	/

“/” represents no research.

**Table 5:** Summary and comparison of silica-based sorbents on adsorption of Sr in lower-acid medium or seawater.

Sorbent	Synthetic Method	Specific Surface Area (m^2^/g)	*K*_d_ Order	Capacity mg Sr(II)/g	Equilibrium Time	Good Adsorption Acidity	Treatment Bed Volume
K_2_Ti_6_O_13_/SiO_2_	sol–gel method	/	/	15	≥8 h	pH: 4.2–6.4	80
Na_2_TinO_2n+1_/SiO_2_	sol–gel method	44.83	Sr^2+^ > Ba^2+^ >> Mg^2+^, Ca^2+^, Cs^+^, K^+^	66.37	<10 min	pH: 3–10	950
h-WO_3_/SiO_2_	hydrothermal method	/	Sr^2+^ > La^3+^ > Mg^2+^ > Dy^3+^, Ca^2+^	9	15 min	pH: 4–7	/
ZrP/MSP	one-pot liquid-phase grafting method	293.73	Sr^2+^ > Ba^2+^ > Ca^2+^ > Cu^2+^ > Mg^2+^	100.77	1.5 h	pH: 4–7	/
Sb_2_O_5_/SiO_2_	vacuum impregnation method	/	Sr^2+^ > Zr^2+^ > Mo^4+^ > Ba^2+^ > La^3+^, Mg^2+^, Dy^3+^, Ca^2+^ (1 M HNO_3_)	160.6	5 min	pH: 6	316
SiMaC	in situ polymerization method	20.8	/	142.5	45 min	pH: 10	/

“/” represents no research.

**Table 6:** Summary and comparison of silica-based sorbents on adsorption of PGM fission products.

Sorbent	PGMs	*K*_d_ Order	Capacity mg	Equilibrium Time	Good (Best) Adsorption Acidity	Column Results	Desorber
(MOTDGA-TOA)/SiO_2_-*P*	Pd, Ru, and Rh	Pd > Zr > Mo > Ru > Rh > La, Ce, Nd, Sm, Gd	About 0.73, 0.31, and 0.63 mmol/g for Ru, Rh, and Pd	2 h for Pd and over 24 h for Ru and Rh/	Best for Pd in 0.1 M HNO_3_, but still kept well in 1–5 M HNO_3_ solution for Pd and Ru	Pd and Ru were separated from Rh, Zr, Mo Re, La, Ce, Nd, Sm, and Gd in 3 M HNO_3_ solution at 323 K.	/
(MOTDGA-Dodecanol)/SiO_2_-*P*	Pd	Pd > Zr > Mo > Ru >> Rh, La, Ce, Nd, Sm, Gd	/	8 h	0.1 M HNO_3_	/	/
(TOA-Dodecanol)/SiO_2_-*P*	Pd	Pd >> Zr, Mo, Ru, Rh, La, Ce, Nd, Sm, Gd	/	8 h	0.1 M HNO_3_	/	/
(Crea+Dodec)/SiO_2_-*P*	Ru, Rh, and Pd	Pd > Ru > Mo > Rh > Zr > Re >> La, Ce, Nd, Sm, Gd	About 0.7 mmol/g for Pd and over 0.35 mmol/g for Ru and Rh	Within 30 min for Pd, about 15 h for Ru, and over 72 h for Rh at 298 K	0.1–5 M HNO_3_ for Pd	Pd and Mo were separated from Ru, Rh, Zr, Re, La, Ce, Nd, Sm, and Gd in 3 M HNO_3_ solution.	/
(Crea+TOA)/SiO_2_-*P*	Ru, Rh, and Pd	Pd > Ru > Rh > Mo > Zr > La, Ce, Nd, Sm, Gd	/	About 24 h	0.1–5 M HNO_3_ for Pd	Pd and partial Ru were separated from Rh, Zr, Mo Re, La, Ce, Nd, Sm, and Gd in 3 M HNO_3_ solution at 323 K.	/
(DAMIA-EH+TOA)/SiO_2_-*P*	Pd and Ru	Pd >> Re >> Ru > Rh, Zr, Mo, Cs, Sr, Ba, La, Ce, Nd, Sm, Eu, Gd	About 0.57 mmol/g for Pd and > 0.3 mmol/g for Ru	10 min for Pd and 5 h for Ru at 298 K	0.1 M HNO_3_ for Pd and 0.5–4 M HNO_3_ for Ru	Pd was separated from Ru, Rh, Zr, Mo Re, Sr, Cs, Ba, La, Ce, Nd, Sm, Eu, and Gd in 3 M HNO_3_ solution at 298 K.	0.01 M SC(NH_2_)_2_ (pH = 2) for Pd
(DAMIA-EH+1-dodecanol)/SiO_2_-*P*	Pd	Pd >> Re > Mo > Zr > Ru	About 0.57 mmol/g for Pd	Within 1 h for Pd	1 M HNO_3_ for Pd	/	/
Me_2_-CA-BTP/SiO_2_-*P*	Pd	Am >> Pd >> Sr, Y, Zr, Ru, Cs, La, Ce, Nd, Sm, Eu, Gd	0.76 mmol/g for Pd	About 12 h	2–4 M HNO_3_ for Pd	/	/
isoHex-BTP/SiO_2_-*P*	Pd	Pd >> Pu, Am >> Sr, Y. Zr, Mo, Tc, Ru, La, Ce, Nd, Sm, Eu, Gd, U	0.85 mmol/g for Pd	About 72 h	2–4 M HNO_3_ for Pd	/	0.5 M SC(NH_2_)_2_ (pH = 1) for Pd
isoBu-BTP/SiO_2_-*P*	Pd, Ru, and Rh	Pd > Ru > Rh <Sr, Y, Zr, Mo, La,Ce, Pr, Nd, Sm, Eu, Gd at 328 K	0.34, 0.33, and 1.06 mmol/g for Ru, Rh, and Pd at 313 K	About 48 and 24 h at 328 K for Pd	0.5–5 M HNO_3_ for Ru, Rh, and Pd	Pd was separated from other fission products during the separation of MA.	0.1 mol/L SC(NH_2_)_2_ (pH = 2) for Pd
TpPa-1/SiO_2_-A600	Pd	Pd >> Sr, Cs, Ba, Ru, Rh, Zr, Mo, Re, La, Ce, Nd, Sm, Eu, Gd	0.12 mmol/g for Pd	About 0.5 h	0.6–5 M HNO_3_	/	0.2 mol/L SC(NH_2_)_2_ (pH = 2) for Pd
dNbpy/SiO_2_-*P*	Pd, Ru, and Rh	Pd > Ru > Rh > Y, La, Ce, Nd, Sm, Eu, Gd	93.0, 46.0, and 14.9 mg/g for Pd, Ru, and Rh	10 min for Pd and 24 h for Ru and Rh	3 M HNO_3_	Pd was separated from Ru, Rh, Y, Sr, Cs, Ba, La, Ce, Nd, Sm, Eu, and Gd in 3 M HNO_3_ solution.	0.1 M HNO_3_ and thiourea
Tp-Azo-COF/SiO_2_	Pd	Pd > Y > Ru > Rh > Pr > La > Nd > Ce > Gd > Sm > Eu	85.4 mg/g for Pd	60 min for Pd	3 M HNO_3_	Pd was separated from Ru, Rh, Ba, Y, Sr, Cs, La, Ce, Nd, Sm, Eu, and Gd in 3 M HNO_3_ solution.	0.1 M HNO_3_ and thiourea
SiAcyl/SiO_2_	Pd	Pd > Mg, Sr, Ni, Co, Ca, Cr, K	81.8 mg/g for Pd	60 min for Pd	1.95–3 M HNO_3_	Pd was separated from Ni, Na, Ca, Mg, and K.	0.1 M HNO_3_ and thiourea
SiVpC/SiO_2_	Pd	Pd > Ru, Rh, Y, La, Ce, Pr, Nd, Sm, Eu, Mo	22.2 mg/g for Pd	2 h for Pd	0.5 M HNO_3_	Pd was separated from Ru, Rh, Ba, Y, Sr, Cs, La, Ce, Nd, Sm, Eu, and Gd in 0.5 M HNO_3_ solution.	0.5 M HNO_3_–0.5 M THU
SBA-15-TEPA	Pd	Pd > Ba > K > Cu > Sr > Na > Zn, Ni, Al, Mg, Ca	84.21 mg/g for Pd	2 h for Pd	1.5 M HNO_3_	Pd was separated from Ni, Ca, Na, Mg, and K.	0.1 M HNO_3_ and thiourea
2AT-SiAaC	Pd	Pd >> Rh, Y, Sr, Ba, Cs, La, Ce, Pr, Nd, Sm, Eu, Gd	62.1 mg/g for Pd	60 min for Pd	0.5 M HNO_3_	Pd was separated from Rh, Y, Sr, Ba, Cs, La, Ce, Pr, Nd, Sm, Eu, and Gd.	0.5 M HNO_3_–0.5 M THU
SiPS-TU	Pd	Pd > Rh, Ru	75.93 mg/g for Pd	1 h	0.1 M HNO_3_	/	/
KNiHC/SiO_2_	Pd	Pd >> Rh, Ru	48.5 mg/g for Pd	About 1 h	1 M NaNO_3_–3 M HNO_3_	/	/

“/” represents no research.

**Table 7:** Summary and comparison of silica-based sorbents on adsorption of Cs(I) in acidic solution.

Sorbent	*K*_d_ Order	Capacity mg Cs(I)/g	Equilibrium Time	Acidity	Column Results	Stability (TOC)	Cs Desorber
(Calix[4] R14+TBP)/SiO_2_-*P*	Cs >> Rb >> K, Na, Sr	/	Within 30 min	4.0 M HNO_3_	Cs and Rb were separated from Ba, Sr, Ru, Fe, K, Na, Mo, Zr, and Pd in 4.0 M HNO_3_.	/	H_2_O
(Calix[4]+MODB)/SiO_2_-*P*	Cs >> Pd, Ru >> La, Y, Mo, Rh, Zr *K*_d_ (Cs) < 50	/	About 30 min	3.0 M HNO_3_	Cs was separated from Pd, La, Y, Mo, Zr, Ru, and Rh in 3.0 M HNO_3_.	/	H_2_O
(Calix[4]+Dodecanol)/SiO_2_-*P*	Cs >> Zr > Mo, Sr, Pd >> La, Nd, Sm, Ga	0.4 mmol Cs/g	5 h	2.0 M HNO_3_	/	≤180 ppm; γ radiation stability was evaluated	/
(Calix[4]+dodecanol+DBS)/SiO_2_-*P*	Cs >> Na, K, Sr, Ru, Rh, Zr, Mo, Y, La, Ce, Eu, Pd	0.12–0.16 mmol Cs/g	More than 60 min	0.5–5 M (0.5.0 best) HNO_3_	/	1 wt% (75ppm) at 318 K	/
BnOCalix[4]C6/SiO_2_-*P*	Cs >> Pd > Rb >> Na, K, Ba, Cs, Y, La, Ru, Mo, Zr	/	More than 60 min	3.0 M HNO_3_	/	about 0.29% (110 ppm)	/
(CalixBNaphC)@SiO_2_-*P*	Cs >> Rb >> K, Fe, Pd, Sr, Fe, Ba	/	About 60 min	3.0 M HNO_3_	Cs and Rb were separated from Li, Na, K, Fe, Sr, Ba, and Pd in 3 M HNO_3_.	/	H_2_O
(Calix[4]+DtBuCH18C6)/SiO_2_-*P*	/	0.15 and 0.24 mmol/g for Cs and Sr	1–2 h for Cs and 1–3 h for Sr	2 and 4 M HNO_3_ for Sr and Cs	Both Sr and Cs can be adsorbed in 3 M HNO_3_ and desorbed by H_2_O.	TOC: 150 ppm	/
AMP/SiO_2_	/	0.36 mmol Cs/g	Within 30 min	over 300 mL/g in 3.0 M HNO_3_	/	good	/

“/” represents no research.

## Data Availability

Data will be made available on request.
